# Hepcidin as a Molecular Hub of Iron Homeostasis: From BMP–SMAD Signaling to Therapeutic Modulation

**DOI:** 10.3390/biom16070947

**Published:** 2026-06-25

**Authors:** Andrea Duminuco, Alessandro Costa, Federica Pilo, Salvatore Scarso, Cesarina Giallongo, Sebastiano Giallongo, Annalisa Santisi, Arianna Sbriglione, Laura Santocono, Giovanni Caocci, Giuseppe A. Palumbo

**Affiliations:** 1 Department of Medical, Surgical Sciences and Advanced Technologies “G.F. Ingrassia”, University of Catania, 95123 Catania, Italy; palumbogiuseppea@gmail.com; 2 Hematology Unit with BMT, A.O.U. Policlinico “G.Rodolico-San Marco”, 95123 Catania, Italy; annalisa_santisi@hotmail.it (A.S.); sbriglionearianna@gmail.com (A.S.); santoconolaura8@gmail.com (L.S.); 3 Hematology and BMT Unit, “A. Businco” Hospital, ARNAS Brotzu, 09121 Cagliari, Italy; alessandro.costa@aob.it (A.C.); federica.pilo@aob.it (F.P.); giovanni.caocci@aob.it (G.C.); 4 Department of Infectious, Tropical Diseases and Microbiology, IRCCS Sacro Cuore Don Calabria Hospital, Negrar di Valpolicella, 37024 Verona, Italy; salvatorescarso31@gmail.com; 5 Department of Biomedical and Biotechnological Sciences, University of Catania, 95123 Catania, Italy; cesarina.giallongo@unict.it; 6 Department of Medicine and Surgery, University of Enna “Kore”, 94100 Enna, Italy; sebastiano.giall@gmail.com

**Keywords:** hepcidin, iron homeostasis, BMP–SMAD signaling, ACVR1/ALK2, β-thalassemia, inflammation, chronic kidney disease, myeloproliferative neoplasms

## Abstract

Hepcidin, a 25-amino-acid peptide hormone produced primarily by hepatocytes, is the master regulator of systemic iron homeostasis. By binding the cellular iron exporter ferroportin and inducing its internalization and lysosomal degradation, hepcidin restricts iron entry into plasma from enterocytes, macrophages, and hepatocytes. Its transcription is governed by an intricate molecular network that integrates iron status, erythropoietic demand, oxygen tension, and inflammation, with the BMP–HJV–ALK2/SMAD axis acting as the canonical activating pathway and erythroferrone (ERFE) and matriptase-2 (TMPRSS6) as physiological suppressors. Dysregulation of hepcidin underpins a wide spectrum of human diseases: insufficient hepcidin drives hereditary hemochromatosis and the iron overload of congenital and acquired ineffective erythropoiesis diseases and other ineffective erythropoiesis syndromes, whereas excessive or inappropriate hepcidin contributes to anemia of inflammation, anemia of chronic kidney disease, iron-restricted erythropoiesis in cancer, the iron-restrictive anemia of myelofibrosis, and pathogen-restrictive nutritional immunity. Within the myeloproliferative neoplasm spectrum, the divergent hepcidin patterns observed in polycythemia vera (suppressed) and myelofibrosis (inappropriately elevated through dual BMP/ACVR1/SMAD and IL-6/STAT3 hyperactivation) exemplify the clinical relevance of this axis and underpin two opposite pharmacologic strategies. Over the past decade, hepcidin pathway pharmacology has matured from proof-of-concept to regulatory milestones, shifting perspectives on several diseases and markedly improving clinical approaches.

## 1. Introduction

Iron is an essential micronutrient involved in oxygen transport, mitochondrial respiration, DNA synthesis, and host defense, yet potentially cytotoxic when in excess through Fenton chemistry and the generation of reactive oxygen species [1]. Mammalian organisms have evolved no regulated mechanism for iron excretion; consequently, the entire systemic balance of body iron (of the order of 3–4 g in the adult human) depends on the tightly controlled absorption of approximately 1–2 mg per day from the duodenum and on the recycling of approximately 20–25 mg per day liberated from senescent erythrocytes by splenic and hepatic macrophages [2,3]. The discovery of hepcidin at the turn of the millennium, independently by groups investigating liver iron-regulated genes and antimicrobial peptides, provided the conceptual unification of this homeostatic system around a single hormonal axis [4,5].

Hepcidin, encoded by the *HAMP* gene on chromosome 19q13.12 and synthesized predominantly by hepatocytes, is a cysteine-rich peptide of 25 amino acids generated by proteolytic maturation from an 84-amino-acid prepropeptide. Its central function is to inhibit cellular iron efflux by binding to ferroportin (SLC40A1), the sole known mammalian iron exporter, leading to both direct inhibition of ferroportin’s iron-export activity and subsequent ubiquitination, internalization, and lysosomal degradation of the transporter [6]. By acting on enterocytes (limiting absorption), on splenic and hepatic macrophages (limiting iron recycling), and on hepatocytes (modulating storage release), hepcidin controls the size of the circulating transferrin-bound iron pool available to the bone marrow activities and peripheral tissues [7].

In the two decades since its identification, hepcidin has moved from a peptide of biochemical curiosity to the molecular hub through which virtually every disorder of iron metabolism can be interpreted, and against which an expanding pharmacological armamentarium is now being deployed. Hereditary hemochromatosis, congenital hemoglobinopathies, anemia of inflammation, anemia of chronic kidney disease, ineffective erythropoiesis in myelodysplastic syndromes, erythrocytosis in polycythemia vera, and the iron-restrictive anemia of myelofibrosis all converge on dysregulation of the hepcidin–ferroportin axis [8,9,10,11]. Among myeloproliferative neoplasms (MPN), the contrast between polycythemia vera (PV), essential thrombocytemia (ET), and myelofibrosis (MF) provides a particularly informative paradigm. In PV, autonomous erythroid proliferation drives erythroferrone-mediated hepcidin suppression and consequent enhanced intestinal iron absorption, which support the erythrocytotic phenotype [12,13]. Therapeutic strategies in PV, therefore, aim to increase effective hepcidin activity through hepcidin-mimetic agents or TMPRSS6 antisense oligonucleotides. In MF, by contrast, the combined hyperactivation of BMP–ACVR1/ALK2–SMAD signaling and interleukin-6 (IL-6)-driven STAT3 activation produces inappropriately elevated hepcidin levels, sequestering iron in the reticuloendothelial system and generating a state of functional iron deficiency superimposed on ineffective erythropoiesis [14,15]. The approval in 2023 of momelotinib (a small-molecule JAK1/JAK2 inhibitor that also uniquely inhibits ACVR1/ALK2) for myelofibrosis with anemia represents the first regulatory milestone for a therapeutic agent that derives its benefit primarily from direct pharmacologic modulation of hepatic hepcidin production [15,16,17,18].

The present review aims to provide a comprehensive, mechanistically anchored, and clinically oriented synthesis of current knowledge on hepcidin.

## 2. Hepcidin: Structure, Synthesis, and Mechanism of Action

### 2.1. Peptide Chemistry and Biosynthesis

Mature human hepcidin-25 is an amphipathic peptide of approximately 2.8 kDa whose tertiary structure is stabilized by four intramolecular disulfide bonds arranged in a ladder configuration. The peptide adopts a hairpin fold organized around an N-terminal region of five residues that is both necessary and sufficient for ferroportin binding and degradation, and a C-terminal region that contributes to overall structural stability [6,19]. Shorter isoforms, hepcidin-22 and hepcidin-20, generated by N-terminal proteolytic processing of the mature peptide, are detectable in plasma and urine, particularly in chronic kidney disease, but lack iron-regulatory activity, consistent with the essential role of the first five amino acids in ferroportin engagement [20].

Hepcidin is produced almost exclusively in the liver, with contributions from extra-hepatic sources, including macrophages, adipocytes, cardiomyocytes (with high hepcidin expression documented in the right atrium), and renal tubular cells, whose systemic relevance appears negligible based on studies in liver-specific Hamp-deficient mice, although local autocrine and paracrine functions in these tissues remain under active investigation [21]. After translation as preprohepcidin, the peptide undergoes signal peptide cleavage and prohormone convertase-mediated processing (predominantly by furin) to generate the bioactive 25-amino-acid form, which is secreted into the bloodstream and circulates partly bound to α2-macroglobulin and albumin [6,20,22]. A notable exception to the predominantly hepatic origin of systemic hepcidin is the central nervous system (CNS), which represents a functionally distinct compartment in iron regulation. The CNS is shielded from systemic iron fluctuations by the blood–brain barrier, and local hepcidin synthesis has been documented in neurons, astrocytes, and microglial cells. Brain-derived hepcidin is thought to regulate iron availability within the CNS independently of the systemic hormonal axis, with proposed roles in neuroprotection, regulation of ferroptosis, and modulation of iron homeostasis in neurodegenerative conditions including Parkinson’s and Alzheimer’s disease, where dysregulated brain iron metabolism is a recognized pathological feature [23,24,25].

### 2.2. Ferroportin: The Molecular Target of Hepcidin

Ferroportin (FPN1, SLC40A1) is a 12-transmembrane domain transporter expressed on the basolateral membrane of duodenal enterocytes, on the plasma membrane of reticuloendothelial macrophages, and on hepatocytes, placental syncytiotrophoblast, renal proximal tubular cells, and, paradoxically, on mature erythrocytes, where its functional role remains under investigation [23]. Binding of hepcidin to ferroportin triggers ubiquitination of a cluster of intracellular lysines, internalization through a clathrin- and adaptor protein 2 (AP-2)-mediated pathway, and degradation predominantly via the lysosomal pathway, with evidence of partial proteasomal co-involvement [24]. Cryo-electron microscopy has more recently delineated the structural basis of the interaction at near-atomic resolution [25]. Hepcidin also appears to function in part as a direct blocker of ferroportin’s iron-transport activity before internalization, consistent with rapid (within minutes) reductions in plasma iron after hepcidin administration [26,27].

From a teleological perspective, this design places the entire systemic iron-flux network under negative hormonal control. High hepcidin sequesters iron in enterocytes (which are subsequently shed) and in macrophages, lowering transferrin saturation and limiting the iron supply to erythroid precursors and tissues. Low hepcidin levels permit intestinal uptake and macrophage iron recycling and release, thereby restoring or increasing transferrin-bound iron and supporting erythropoiesis [28]. Hepcidin overexpression mimics functional iron deficiency even in the presence of adequate body iron storage (ferritin level), while hepcidin insufficiency promotes enhanced intestinal iron absorption and altered iron distribution which, under normal dietary iron intake, progressively result in parenchymal iron accumulation (the hallmark of hereditary hemochromatosis), congenital hemoglobinopathies and myelodysplastic syndrome [29].

## 3. Molecular Regulation of Hepcidin Expression

Hepcidin transcription is governed by at least four converging input signals (iron status, erythropoietic activity, oxygen tension, and inflammation) that integrate at the hepatocyte level through partially overlapping intracellular signaling pathways. Disentangling these inputs has been one of the most productive areas of iron biology in the past fifteen years.

### 3.1. The BMP–SMAD Axis and the Central Role of ACVR1/ALK2

The dominant iron-sensing pathway upstream of hepcidin is the bone morphogenetic protein (BMP)–SMAD signaling cascade. Liver sinusoidal endothelial cells (LSECs) sense iron through still incompletely understood mechanisms (the iron-responsive elements of BMP6 transcription itself, the involvement of TFR1 cycling, and intracellular iron pools have all been implicated) and respond by secreting BMP6, with BMP2 contributing in a parallel manner to basal hepcidin expression [30,31]. BMP ligands engage type I and type II BMP receptors on the hepatocyte surface; the type I receptors ALK2 (encoded by *ACVR1*) and ALK3 (encoded by *BMPR1A*) are the principal mediators of iron-dependent and basal hepcidin induction, respectively. The co-receptor hemojuvelin (HJV, encoded by *HFE2*) is essential for signal amplification, and loss-of-function mutations in HJV produce the severe juvenile form of hereditary hemochromatosis [8,32].

Receptor engagement leads to phosphorylation of the receptor-regulated SMADs (SMAD1, SMAD5, SMAD9), which heterodimerize with the common mediator SMAD4 and translocate to the nucleus to transactivate the *HAMP* promoter through binding to two evolutionarily conserved BMP-responsive elements (BMP-RE1 and BMP-RE2) [32]. The functional asymmetry between ALK2 and ALK3 has been clarified by murine genetic studies and by the characterization of the immunophilin FKBP12, which physically constrains ALK2 in an inactive state under basal conditions and whose disinhibition (e.g., during iron loading) permits maximal BMP6-driven signaling [33,34]. The clinical relevance of ACVR1 extends well beyond iron biology: gain-of-function mutations of *ACVR1*, notably R206H, cause fibrodysplasia ossificans progressiva, while activin A-induced aberrant ALK2 signaling is the basis for the use of activin trap ligand in ineffective erythropoiesis diseases [35]. Crucially, ACVR1/ALK2 has emerged as the molecular target of momelotinib in myelofibrosis [14,15].

The HFE–TFR2–HJV protein complex on the hepatocyte plasma membrane functions as an iron-sensing platform that modulates BMP–SMAD signaling. Mutations in HFE (most commonly p.C282Y in homozygous configuration), TFR2, and HJV all converge on attenuated hepcidin transcription and lead to hepcidin-insufficient hereditary hemochromatosis [36]. Despite decades of investigation, the precise molecular mechanism by which hepatocytes sense circulating iron and translate this signal into graded hepcidin transcription remains incompletely understood and represents one of the central unresolved questions in iron biology. Current models propose that diferric transferrin (Tf-Fe_2_) competes with HFE for binding to TFR1 on the hepatocyte surface; at high iron concentrations, elevated Tf-Fe_2_ displaces HFE from TFR1, allowing HFE to potentiate BMP receptor signaling, while holotransferrin binding stabilizes TFR2 and promotes its interaction with the BMP receptor complex, thereby sensitizing hepatocytes to BMP-driven hepcidin induction [37,38]. Recent work has refined this model. Hepatocyte TFR1 has been shown to fine-tune hepcidin expression in an HFE-dependent manner according to hepatocellular iron load, and diferric transferrin regulates TFR2 protein stability post-translationally rather than transcriptionally [39,40]. The precise structural interactions through which these sensors drive BMP signaling and graded hepcidin transcription remain incompletely understood [41]. At high iron concentrations, elevated Tf-Fe_2_ displaces HFE from TFR1, freeing HFE to interact with TFR2 and HJV in a trimeric complex that amplifies BMP–SMAD signaling toward the HAMP promoter [38]. In parallel, iron sensing by liver sinusoidal endothelial cells (LSECs) drives BMP6 transcription through a now substantially clarified mechanism. During iron overload, LSECs internalize non-transferrin-bound iron (NTBI), which generates mitochondria-derived oxidative stress and activates the NRF2 transcription factor, in turn inducing BMP6 expression, whereas TFR1-mediated holotransferrin uptake contributes mainly under low-iron conditions [42,43,44,45]. The relative contributions of these mechanisms and their dynamic integration under physiological versus pathological iron-loading conditions remain active areas of investigation [3]. Together, these observations establish the BMP–SMAD axis, with ACVR1/ALK2 at its center, as both the dominant iron-sensing module of hepcidin regulation and a high-value target for pharmacologic modulation in opposite directions depending on the clinical context.

### 3.2. Erythroferrone and Erythroid Suppression of Hepcidin

The physiological need to suppress hepcidin during compensatory erythropoiesis (whether after blood loss, hypoxia, or pharmacologic erythropoietin [EPO] administration) was long suspected to depend on a soluble erythroid factor before erythroferrone (ERFE, encoded by *FAM132B*/*ERFE*) was identified by Kautz and colleagues. ERFE is a member of the C1q–TNF-related protein family secreted by EPO-stimulated erythroblasts via STAT5-dependent transcription; it acts on the liver to repress hepcidin in concert with reduced BMP signaling [46]. Mechanistic studies have shown that ERFE acts at least in part by binding and sequestering BMP2, BMP5, BMP6, and BMP2/6 heterodimers, thereby reducing BMP availability at the hepatocyte surface [47,48].

In disorders of ineffective erythropoiesis, such as congenital dyserythropoietic anemias, and congenital or acquired sideroblastic anemias, ERFE is chronically and markedly upregulated. In murine models, ERFE ablation in *Hbb^Th3/+^* thalassemic mice restores hepcidin to near-normal levels and reduces iron overload without ameliorating anemia, demonstrating that ERFE is a major driver of pathological hepcidin suppression in this setting [49]. ERFE also contributes to recovery from anemia of inflammation, but its capacity to suppress hepcidin under strong inflammatory pressure is, however, blunted [50]. Importantly for the MPN, ERFE elevation is the dominant driver of hepcidin suppression in PV, but is substantially less prominent in MF, where erythroid expansion is constrained by marrow fibrosis and where the inflammatory milieu predominates [51]. Beyond iron, ERFE has emerged as a modulator of bone remodeling by sequestering BMP, providing a mechanistic link between ineffective erythropoiesis and the bone disease that complicates thalassemia syndromes [52].

### 3.3. Matriptase-2 (TMPRSS6) and the Negative Feedback Loop

Matriptase-2, a type II transmembrane serine protease encoded by *TMPRSS6* and expressed predominantly in the liver, exerts a powerful inhibitory effect on hepcidin by reducing the efficiency of BMP–SMAD signaling. In vitro studies have demonstrated that TMPRSS6 can cleave hemojuvelin at the hepatocyte surface [53]. It should be noted that this cleavage has not been directly demonstrated in vivo, and TMPRSS6-deficient mice paradoxically show decreased HJV protein levels, suggesting more complex regulatory mechanisms [54]. Furthermore, studies showing that catalytically inactive matriptase-2 mutants retain their hepcidin-suppressor function suggest that the inhibitory activity of TMPRSS6 is not exclusively dependent on its proteolytic activity, and that scaffold or protein–protein interaction mechanisms may also contribute [55]. Loss-of-function mutations in *TMPRSS6* cause iron-refractory iron deficiency anemia (IRIDA), an autosomal recessive disorder characterized by microcytic anemia with low transferrin saturation, inappropriately elevated hepcidin, and poor response to oral and even, in part, intravenous iron supplementation [56]. From a translational perspective, TMPRSS6 is a particularly attractive pharmacologic target precisely because of this physiology. Pharmacologic inhibition of matriptase-2 raises endogenous hepcidin and restricts iron availability, which is the rationale for the antisense oligonucleotide sapablursen [57].

More recent work has uncovered an additional regulatory layer in which the immunophilin FKBP12 and TMPRSS6 interact functionally to constrain BMP signaling. Antisense-mediated downregulation of Fkbp12 in mice is accompanied by reciprocal upregulation of Tmprss6, suggesting a compensatory braking mechanism that fine-tunes hepatocyte BMP responsiveness [58].

### 3.4. Inflammation: IL-6/STAT3 and the Hepcidin Response

Inflammatory induction of hepcidin is mediated principally by IL-6 through binding to the IL-6 receptor on hepatocytes, JAK2-mediated phosphorylation of STAT3, and STAT3 binding to a specific STAT3-responsive element in the *HAMP* promoter [59]. Other cytokines, including IL-22 and activin B, can also induce hepcidin, and the IL-6/STAT3 pathway shows extensive crosstalk with the BMP–SMAD axis. Full inflammatory hepcidin induction requires a basal level of BMP signaling, and BMP–SMAD signaling is in turn modulated by inflammatory inputs [59,60]. Toll-like receptor 4 (TLR4) activation by lipopolysaccharide (LPS) can also induce hepcidin through MyD88-dependent mechanisms in macrophages and hepatocytes, providing a more direct innate-immune induction pathway [10,60].

From a clinical standpoint, this inflammatory induction is the molecular basis of anemia of inflammation (also termed anemia of chronic disease), one of the most common anemias encountered in clinical practice. Chronic IL-6-driven elevation of hepcidin causes both reduced intestinal iron absorption and macrophage iron sequestration, generating the characteristic biochemical signature of low serum iron, low transferrin saturation, and normal-to-high ferritin (a state of functional iron deficiency superimposed on adequate or even excessive total body iron stores) [7,59]. This molecular pattern is also dominant in myelofibrosis, where IL-6 elevation is one half of a dual mechanism (with BMP6/ACVR1 hyperactivation) that drives inappropriate hepcidin elevation [61].

### 3.5. Oxygen and Erythropoietic Sensing

**Figure 1:**
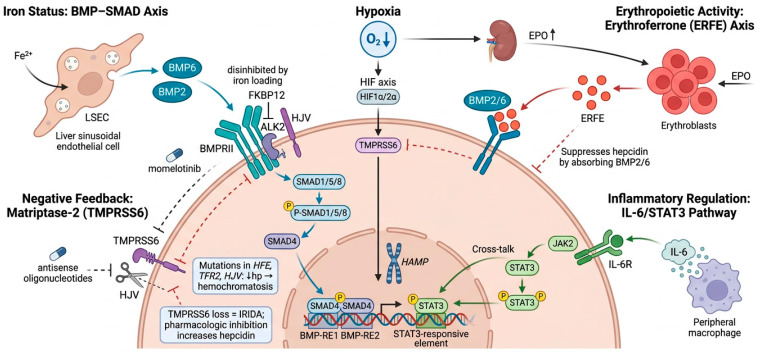
Molecular regulation of hepcidin expression in hepatocytes. HAMP: Hepcidin antimicrobial peptide gene; LSEC: Liver sinusoidal endothelial cell; BMP = Bone morphogenetic protein; BMPRII/ALK2/ALK3 = BMP receptors; HJV: Hemojuvelin; SMAD: Mothers against decapentaplegic homolog; FKBP12: FK506-binding protein 12; ERFE: Erythroferrone; EPO: Erythropoietin; HIF: Hypoxia-inducible factor, TMPRSS6: Matriptase-2; IRIDA: Iron-refractory iron deficiency anemia; IL-6: Interleukin-6; IL-6R: IL-6 receptor; JAK2: Janus kinase 2 Interleukin-6; STAT3: Signal transducer and activator of transcription 3.

Hypoxia suppresses hepcidin expression through both direct and indirect mechanisms. The HIF (hypoxia-inducible factor) system has been shown in vitro to contribute to transcriptional upregulation of *TMPRSS6* through HRE elements in its promoter, even if the effect of hypoxia on TMPRSS6 protein levels and activity in vivo has not been formally demonstrated, and this pathway should therefore be considered as a candidate mechanism pending in vivo validation [62]. The dominant effect, however, is indirect and mediated through the EPO–erythroblast–ERFE axis: hypoxia drives renal EPO production, which in turn stimulates erythroblast proliferation and ERFE secretion, suppressing hepcidin and mobilizing iron for new red cell production [63]. The hierarchical primacy of these signals and their interactions with concurrent iron status and inflammation define the integrative logic of hepcidin regulation (Figure 1).

## 4. Hepcidin Dysregulation in Human Disease

Disorders of the hepcidin–ferroportin axis follow a unifying principle: insufficient hepcidin produces iron overload and tissue-related damage, whereas excessive or inappropriately elevated hepcidin produces iron-restricted erythropoiesis with relative tissue iron sparing in macrophages.

### 4.1. Iron Overload Disorders: Hereditary Hemochromatosis and Thalassemias

#### 4.1.1. Hereditary Hemochromatosis

Hereditary hemochromatosis (HH) is the prototypical disorder of primary hepcidin insufficiency. The most common form, *HFE*-related HH (type 1, OMIM #235200), is caused by homozygosity for the p.C282Y variant of *HFE* and, in the European-descent population, has a carrier frequency of approximately 1 in 10 and a homozygous prevalence of 1 in 200–400, although clinical penetrance is remarkably low and highly variable: population-based studies estimate that clinically significant organ damage develops in approximately 10–30% of male homozygotes and in fewer than 5% of female homozygotes over a lifetime, with the majority remaining asymptomatic or minimally affected. This low penetrance, modulated by sex, dietary iron intake, alcohol consumption, blood donation history, and genetic modifiers, has profound implications for population screening strategies and counseling of incidentally identified C282Y homozygotes [36,38]. Importantly, despite this low penetrance, large population-based cohort and genome-wide association studies have demonstrated that C282Y homozygosity is associated with increased risk of several outcomes, including liver disease and hepatocellular carcinoma, diabetes, musculoskeletal morbidity (arthropathy and joint replacement), and increased all-cause morbidity, even in individuals with normal iron indices at enrollment [64,65,66,67,68]. Less common forms include juvenile HH (type 2A from biallelic *HFE2* mutations and type 2B from *HAMP* mutations), *TFR2*-related HH (type 3), and ferroportin disease (type 4), the latter being further subdivided into a loss-of-function form (4A, with macrophage iron sequestration and mild anemia) and a hepcidin-resistant gain-of-function form (4B, indistinguishable from classical HH in phenotype) [69]. A revised nomenclature proposed by the BioIron Society reclassifies these disorders based on the causative gene rather than numerical type designation (HFE-, HJV-, HAMP-, TFR2-, and SLC40A1-hemochromatosis, respectively), providing a more intuitive molecular framework [70].

The molecular common denominator of types 1–3 is reduced BMP–SMAD signaling to hepcidin in the face of elevated iron stores. The iron sensor is biochemically blind, hepcidin remains inappropriately low relative to iron burden, intestinal absorption is unchecked, and macrophage iron recycling proceeds unrestrained, leading to progressive parenchymal accumulation in liver, heart, pancreas, anterior pituitary, and synovium [71]. The clinical translation of this molecular understanding has been the recognition that hepcidin replacement or BMP pathway agonism are mechanistically rational therapeutic strategies, currently in preclinical and early clinical development, though it must be noted that in patients with established hemochromatosis, who are typically iron-overloaded at diagnosis, these approaches cannot replace phlebotomy as primary iron-depletion therapy. Their potential role lies rather in maintenance after iron stores have been normalized, or as preventive intervention in genetically identified individuals prior to the development of overt overload [72].

#### 4.1.2. β-Thalassemia and Ineffective Erythropoiesis

β-thalassemia exemplifies the iron overload that arises from the combination of ineffective erythropoiesis and hepcidin suppression. The imbalance between α- and β-globin chain synthesis leads to apoptosis of erythroid precursors in the bone marrow, expansion of erythropoiesis, and marked elevation of ERFE; the resulting chronic hepcidin suppression drives accelerated dietary iron absorption and macrophage iron release, producing iron overload even in non-transfusion-dependent thalassemia (NTDT) [49,52]. In transfusion-dependent thalassemia (TDT), transfusional iron loading is superimposed on this hyperabsorptive state, although transfusions themselves transiently restore hepcidin by suppressing endogenous erythropoiesis and ERFE [73].

From a therapeutic standpoint, this molecular framework supports three complementary strategies, starting from the reduction of ineffective erythropoiesis, to the pharmacological restriction of iron availability through hepcidin mimetics or ferroportin inhibitors (vamifeport, rusfertide), and upstreaming the modulation of hepcidin via TMPRSS6 inhibition (sapablursen, although clinical development in β-thalassemia has been discontinued) [74,75].

### 4.2. Anemia of Inflammation and the Iron–Inflammation Interface

Anemia of inflammation (AI), historically anemia of chronic disease (ACD), is a multifactorial condition in which hepcidin-driven functional iron restriction represents the dominant pathophysiological mechanism. Concurrent contributors include cytokine-mediated suppression of erythroid progenitor proliferation, impaired endogenous EPO production, reduced erythrocyte survival, and blunted bone marrow responsiveness to EPO [76,77]. Sustained IL-6 elevation in chronic infections, autoimmune disorders, malignancy, and chronic kidney disease maintains hepcidin transcription, sequestering iron in macrophages and reducing intestinal absorption. The resulting functional iron deficiency limits erythroid iron supply despite repleted body iron stores. The biochemical signature (low serum iron, low transferrin, low transferrin saturation, normal-to-high ferritin, increased C-reactive protein) is the clinical translation of this molecular state [7,78].

Serum hepcidin measurement, when standardized, can in principle help distinguish absolute iron deficiency (low hepcidin) from functional iron deficiency due to inflammation (high hepcidin), and to predict response to iron therapy. However, the lack of internationally harmonized assays, the wide biological inter-individual variability, and substantial diurnal variation have limited routine clinical adoption [20,78]. Anti-hepcidin and anti-BMP6 strategies have undergone clinical evaluation in inflammatory anemias with mixed results, reflecting the complex multifactorial nature of these conditions [8].

### 4.3. Anemia of Chronic Kidney Disease and the Cardio-Renal Axis

Anemia is highly prevalent in chronic kidney disease (CKD), arising from a combination of relative erythropoietin deficiency, iron-restricted erythropoiesis, chronic inflammation, uremic toxin-mediated marrow suppression, and shortened red cell survival [79]. Hepcidin is centrally involved in the iron-restrictive component: serum hepcidin-25 is elevated in CKD, particularly in dialysis-dependent patients, due to both inflammatory upregulation and reduced renal clearance [80,81]. Hepcidin contributes to the so-called erythropoiesis-stimulating agent (ESA) hyporesponsiveness that characterizes a substantial subset of CKD patients [81,82].

Beyond its role in anemia, hepcidin has been investigated as a candidate cardiovascular biomarker in CKD. Elevated hepcidin levels have been associated with macrophage iron accumulation in atherosclerotic plaques, oxidative stress, and adverse outcomes, including all-cause mortality and progression to end-stage renal disease in diabetic CKD cohorts [83,84]. Whether hepcidin modulation will translate into cardiovascular benefit in CKD remains to be established. The recent identification of a positive association between elevated ERFE, hepcidin, and EPO with anemia severity in CKD cohorts supports the relevance of this axis as both a biomarker and a potential therapeutic target [85].

In parallel, hepcidin elevation in heart failure has been linked to the highly prevalent functional iron deficiency observed in this population, which contributes to symptom burden and reduced exercise tolerance and is the target of intravenous iron therapy [86]. The relationship between hepcidin and iron status in heart failure is complex and influenced by inflammation, congestion, and concurrent renal dysfunction [87].

### 4.4. Myeloproliferative Neoplasms: A Tale of Two Opposite Hepcidin Phenotypes

The classical Philadelphia-negative MPN (PV, ET and MF), share a common molecular biology centered on constitutive activation of JAK-STAT signaling through JAK2, CALR, or MPL driver mutations [88,89]. Despite this shared pathogenetic backbone, they display strikingly divergent patterns of iron metabolism that map cleanly onto the hepcidin regulatory framework and, accordingly, demand opposite therapeutic strategies.

#### 4.4.1. Polycythemia Vera: Hepcidin Suppression and Iron-Restrictive Therapy

In PV, autonomous EPO-independent erythropoiesis sustains erythroblast proliferation and consequent ERFE-mediated hepcidin suppression, thereby enhancing intestinal iron absorption and supporting the erythrocytotic phenotype [90]. The clinical mainstay of PV management (therapeutic phlebotomy targeting a hematocrit below 45%) capitalizes on iron restriction as an indirect cytoreductive strategy to reduce the thrombotic risk, but achieves it at the cost of iatrogenic systemic iron deficiency and its associated symptoms (fatigue, restless legs, cognitive complaints), which contribute substantially to the disease symptom burden [91,92,93,94]. From a mechanistic perspective, restoration of effective hepcidin–ferroportin signaling represents a physiologically rational strategy to control erythrocytosis without iatrogenic systemic iron deficiency, and underlies the development of the hepcidin mimetic rusfertide and the TMPRSS6 antisense agent sapablursen, both of which raise effective hepcidin activity and restrict iron availability to erythroid precursors [95,96,97].

#### 4.4.2. Myelofibrosis: Dual BMP6/ACVR1 and IL-6/STAT3 Hepcidin Overproduction

Myelofibrosis presents the opposite biochemical and pharmacologic problem to PV. Despite sharing the JAK-STAT activation that drives PV, MF is characterized by (often severe and progressive) anemia, transfusion dependence, marrow fibrosis with extramedullary hematopoiesis, splenomegaly, and a heavy constitutional symptom burden [88]. Hepcidin is inappropriately elevated in most MF patients, sequestering iron in the reticuloendothelial system and generating a state of iron-restricted erythropoiesis [15]. The molecular basis is now recognized as dual.

First, BMP6–ACVR1/ALK2–SMAD signaling is aberrantly hyperactivated in MF, with elevated serum BMP6 levels and increased hepatic ACVR1 signaling activity driving constitutive hepcidin transcription independently of body iron status [98]. Second, MF is the MPN with the most pronounced inflammatory cytokine signature, including elevated levels of IL-6, TNF-α, IL-8, and IL-1RA, and it synergizes with the BMP–SMAD branch [14,97]. Compared with β-thalassemia or PV, the ERFE-mediated suppressive signal is attenuated in MF because erythroid expansion is constrained by progressive marrow fibrosis, so the dominant input on hepcidin is upward rather than downward. The result is a phenotype of high or inappropriately normal hepcidin, high or normal ferritin (further increased by transfusions), and low transferrin saturation, with functional iron-restricted anemia that responds poorly to ESA or oral iron and that, in transfused patients, coexists with reticuloendothelial iron overload [14,15].

This molecular framework has direct therapeutic implications. Ruxolitinib, although clinically effective on splenomegaly and constitutional symptoms via JAK1/JAK2 inhibition, has no inhibitory activity on ACVR1/ALK2 and does not directly suppress hepcidin [99]. The modest anemia improvement observed in a subset of patients after the first months of ruxolitinib is largely attributed to reduced IL-6-driven inflammation rather than to a direct hepcidin effect [14,100,101]. By contrast, momelotinib (a JAK1/JAK2 inhibitor that uniquely inhibits ACVR1/ALK2 at clinically relevant concentrations) targets both arms of the hepcidin-inducing pathway in MF. It directly suppresses BMP/ACVR1/SMAD signaling in hepatocytes and concurrently dampens IL-6/JAK/STAT3 signaling, thereby reducing hepatic hepcidin production and alleviating iron-restricted erythropoiesis. Pacritinib, another JAK2/IRAK1/ACVR1 inhibitor, shares part of this mechanistic profile and is FDA-approved for MF with severe thrombocytopenia [102].

### 4.5. Hepcidin, Infection, and Nutritional Immunity

From an evolutionary perspective, hepcidin originated as an antimicrobial peptide of the defensin family before acquiring its hormonal iron-regulatory function; its induction during infection is best understood as a form of nutritional immunity, the deliberate sequestration of host iron to deprive invading pathogens of an essential micronutrient [9,10]. Acute hypoferremia induced by hepcidin is observed across a wide range of bacterial, fungal, and protozoal infections and may exert protective effects against extracellular siderophilic pathogens such as *Vibrio vulnificus*, *Yersinia species*, and some *Mycobacterium tuberculosis* settings [10]. Conversely, by promoting intracellular iron retention in macrophages, sustained hepcidin elevation may favor the persistence of intracellular pathogens such as *Salmonella*, *Mycobacterium*, and *Plasmodium*, particularly in their hepatic and erythrocytic stages [9].

Clinically, this duality has direct implications for the management of iron supplementation in malaria-endemic regions, where indiscriminate iron supplementation in children has been associated with increased risk of severe infectious morbidity, and for the design of host-directed antimicrobial therapies aimed at modulating iron availability. Recent murine and translational work has also linked hepcidin-mediated hypoferremia to impaired adaptive immune responses, including blunted vaccine responses, suggesting that the iron–infection axis is bidirectional and that excessive nutritional immunity may itself have immunological costs [103].

## 5. Hepcidin as a Biomarker: Diagnostic and Prognostic Considerations

Despite a decade of investigation, hepcidin has not yet entered routine clinical laboratory use, primarily because of pre-analytical and analytical challenges. Current quantification methods include immunoassays (ELISA, competitive ELISA) using polyclonal or monoclonal antibodies that variably cross-react with hepcidin isoforms, and mass spectrometry-based assays (SELDI-TOF, LC-MS/MS) that can selectively measure hepcidin-25 [104]. The Hepcidin Round Robin and other international harmonization efforts have made progress toward inter-laboratory comparability, but reference intervals remain method-dependent, and substantial diurnal variation (with morning peaks) and post-prandial effects complicate single time-point interpretation [20,105].

Conceptually, hepcidin measurement could refine the differential diagnosis of iron-restricted anemia (low hepcidin: iron deficiency anemia; high hepcidin: IRIDA or anemia of inflammation), predict response to oral iron in patients with mixed inflammatory and absolute iron deficiency, and guide intravenous iron therapy in inflammatory states and CKD [20]. The hepcidin/ferritin ratio and hepcidin-based decision algorithms have shown promise in research settings but require prospective validation [78]. Hepcidin elevation in CKD has shown association with mortality and progression to ESRD in diabetic cohorts, supporting its potential prognostic value beyond anemia management [84]. In MPNs, hepcidin and ERFE measurements are emerging as candidate pharmacodynamic biomarkers for the assessment of response to hepcidin-targeted therapies (rusfertide and sapablursen in PV) and for the prediction of anemia response to momelotinib in MF, with translational studies showing dynamic reductions in serum hepcidin upon ACVR1/ALK2 inhibition that parallel transfusion independence responses [96,97,106].

## 6. Therapeutic Modulation of the Hepcidin–Ferroportin Axis

The translational pipeline targeting the hepcidin–ferroportin axis has expanded considerably over the past decade, from preclinical proof-of-concept studies to approved therapies and pivotal phase 3 trials. The conceptual rationale can be subdivided into two broad strategies, raising effective hepcidin activity for conditions of iron overload or unwanted erythrocytosis, or lowering hepcidin or antagonizing its action for anemia of inflammation, anemia of CKD, and for the iron-restrictive anemia of myelofibrosis.

**Figure 2:**
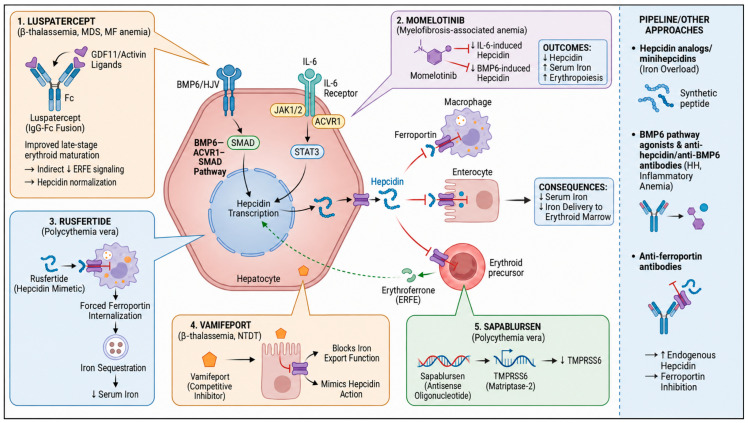
Therapeutic approaches targeting the hepcidin–ferroportin axis.

The therapeutic landscape is summarized in Figure 2.

### 6.1. Luspatercept: Activin Receptor IIB Ligand Trap for Ineffective Erythropoiesis

Luspatercept (formerly ACE-536) is a recombinant fusion protein consisting of a modified extracellular domain of activin receptor type IIB (ActRIIB) linked to the human IgG1 Fc domain. By acting as a ligand trap for selected TGF-β superfamily members, notably activin B and other ActRIIB ligands, murine studies suggest that GDF11 is unlikely to be the primary mediator of its erythropoietic effect; it relieves a SMAD2/3-mediated brake on late-stage erythroid maturation, thereby increasing red cell production while reducing ineffective erythropoiesis [107]. Mechanistically, this attenuates erythroblast apoptosis and ERFE secretion, thereby indirectly normalizing the hepcidin–ferroportin axis [75].

In the pivotal phase 3 BELIEVE trial, 336 adults with transfusion-dependent β-thalassemia were randomized 2:1 to luspatercept or placebo; a ≥33% reduction in transfusion burden (with reduction of at least 2 RBC units) during weeks 13–24 was achieved by 21.4% of patients on luspatercept versus 4.5% on placebo (*p* < 0.001), with consistent benefit across subgroups [108]. The drug was approved for adult β-thalassemia. In transfusion-dependent low-risk and intermediate-1 MDS, the COMMANDS trial established luspatercept as first-line ESA-naive therapy with superior transfusion independence rates compared to epoetin alfa, broadening its label [109]. Luspatercept is also under evaluation in MF-associated anemia (INDEPENDENCE phase 3 trial in transfusion-dependent MF on stable JAK inhibitor therapy). Adverse events observed across these programs include bone pain, arthralgia, fatigue, and a modest signal for thromboembolic events, which warrants monitoring in high-risk populations.

### 6.2. Momelotinib: The JAK1/JAK2/ACVR1 Inhibitor and the First Approved Direct Hepcidin-Pathway Modulator

Momelotinib is an oral small-molecule kinase inhibitor with a uniquely integrated mechanism of action on the hepcidin axis. In addition to inhibiting JAK1 and JAK2, providing spleen, symptom, and partial anemia benefits typical of the JAK-inhibitor class, momelotinib directly inhibits ACVR1/ALK2 at clinically relevant concentrations, thereby suppressing hepatic hepcidin production through the BMP6–ACVR1–SMAD pathway, while its JAK1/2 activity concurrently dampens IL-6-driven hepcidin induction via STAT3. The combined effect is reduced serum hepcidin, increased iron availability for erythropoiesis, and clinical improvement of the iron-restricted anemia of MF [14,18].

Preclinical validation showed in a rat model of anemia of chronic disease that momelotinib normalized hemoglobin and red cell counts through direct ACVR1/ALK2 inhibition and consequent suppression of hepatocyte hepcidin, an effect not observed with ruxolitinib at equivalent doses. Clinical translation followed in a translational phase 2 study in transfusion-dependent MF, in which momelotinib reversed transfusion dependency in 41% of patients (95% CI 26–58%) and produced measurable reductions in serum hepcidin paralleling the hematologic responses [106].

The pivotal randomized phase 3 evidence comes from two complementary trials. SIMPLIFY-1 compared momelotinib with ruxolitinib in JAK-inhibitor-naïve MF and demonstrated a non-inferior splenic response with superior transfusion independence rates, favoring momelotinib, although the symptom response endpoint did not meet non-inferiority [110]. SIMPLIFY-2 enrolled patients previously exposed to ruxolitinib but did not meet its primary endpoint of spleen volume reduction versus best available therapy, although secondary anemia endpoints favored momelotinib [111]. The double-blind MOMENTUM phase 3 trial randomized 195 symptomatic, anemic MF patients previously treated with a JAK inhibitor to momelotinib 200 mg daily versus danazol 600 mg daily. Momelotinib met all key endpoints, with a TSS50 response at week 24 of 25% versus 9% (*p* = 0.0095), a spleen response ≥35% in 23% versus 3%, and transfusion-independence response in 31% versus 20%, with a favorable safety profile [112,113]. On the basis of MOMENTUM, momelotinib received approval for the treatment of MF with anemia, in both JAK-inhibitor-naïve and JAK-inhibitor-experienced patients [114].

From a mechanistic and translational standpoint, momelotinib represents the most direct clinical validation of the hepcidin–ACVR1–SMAD axis as a therapeutic target. Its dual JAK1/2 and ACVR1 activity uniquely addresses the dual molecular driver of MF anemia (BMP6/ACVR1 plus IL-6/STAT3 hepcidin overproduction) and explains why it confers anemia benefits not seen with ruxolitinib, with reduced risk of immunosuppression compared to ruxolitinib [115,116,117,118,119,120,121,122,123]. Beyond MF, this mechanism rationally extends to other states of dual inflammatory and BMP–SMAD-driven hepcidin elevation, including anemia of inflammation in autoimmune and oncologic settings and possibly anemia of CKD, although clinical evidence in these contexts remains preclinical or early-phase. The principal adverse events of momelotinib include thrombocytopenia, anemia (early on-treatment dip before improvement), diarrhea, and peripheral neuropathy, the latter being a distinctive class effect that requires monitoring and rare cases of hepatic enzyme elevation and infection have also been reported [114,124,125].

### 6.3. Rusfertide: Hepcidin Mimetic Peptide for Polycythemia Vera

Rusfertide (PTG-300) is an injectable peptide mimetic of hepcidin designed to bind ferroportin and recapitulate the iron-restrictive effects of the endogenous hormone. Its primary clinical development has been in PV, where the goal is to achieve durable hematocrit control without phlebotomy and with reduced symptom burden [95].

The phase 2 REVIVE trial in phlebotomy-dependent PV demonstrated robust efficacy. In part 1 (28-week open-label dose-finding), rusfertide reduced phlebotomy need and stabilized hematocrit; in part 2 (12-week randomized withdrawal), the response rate (hematocrit control without phlebotomy) was 69% with rusfertide versus 19% with placebo (*p* < 0.001) [95]. The pivotal phase 3 VERIFY trial enrolled 293 patients with PV and met its primary endpoint. At the 32-week analysis, the response rate during weeks 20–32 was 76.9% with rusfertide plus standard of care versus 32.9% with placebo plus standard of care (*p* < 0.0001), with benefit consistent across PV risk strata and concomitant cytoreductive therapy [97]. Updated 52-week data confirmed the durability of response, with 61.9% of continuously treated patients maintaining the absence of phlebotomy eligibility through week 52 [126]. The principal toxicities reported are injection-site reactions and hyperpigmentation. Longer follow-up is needed to define the durability of benefit and long-term safety, including thrombotic events, malignancy signals, and disease transformation.

### 6.4. Vamifeport: First-in-Class Oral Ferroportin Inhibitor

Vamifeport (VIT-2763) is a small-molecule inhibitor of ferroportin that competes with hepcidin for binding to the transporter, recapitulating, with slower kinetics and somewhat lower efficiency, the hepcidin-induced reduction in cellular iron efflux [127,128]. The molecular mechanism has been confirmed by cryo-electron microscopy structures showing vamifeport bound to ferroportin in distinct outward-facing and occluded conformations [25]. In murine models of β-thalassemia (*Hbb^Th3/+^*), vamifeport reduced ineffective erythropoiesis, splenomegaly, and non-transferrin-bound iron formation, both as monotherapy and in combination with transfusions [129]. In a 12-week, randomized, double-blind, placebo-controlled phase 2a trial in adults with NTDT, vamifeport demonstrated a favorable safety and tolerability profile comparable to placebo and produced the expected pharmacodynamic effects (reduction in serum iron, decreased transferrin saturation, decreased non-transferrin-bound iron), without, however, achieving clinically meaningful hemoglobin increases in this short-duration study [130]. Oral administration provides a substantial practical advantage over injectable agents, particularly for pediatric and chronic-use applications.

### 6.5. Sapablursen: Antisense Oligonucleotide TMPRSS6 Inhibitor

Sapablursen is a GalNAc-conjugated, hepatocyte-targeted antisense oligonucleotide that reduces hepatic TMPRSS6 mRNA and thereby increases endogenous hepcidin production. This strategy is mechanistically rational for any condition characterized by inappropriately low hepcidin and benefiting from increased iron sequestration, with PV being the lead indication [131,132]. Initial development in β-thalassemia was discontinued due to insufficient clinical benefit in phase 2 trials, illustrating the imperfect translation of preclinical rationale into hematological efficacy in this complex disorder [133]. Development has continued in PV, where the phase 2a IMPRSSION trial (NCT05143957), enrolling 49 phlebotomy-dependent PV patients across multiple dose cohorts, met its primary objective, demonstrating dose- and time-dependent increases in serum hepcidin with corresponding reductions in hematocrit and trends toward improvement in MPN-SAF Total Symptom Score [131,134].

### 6.6. Other Strategies and the Broader Pipeline

Beyond the five agents discussed above, the hepcidin pipeline includes several additional strategies at varying stages of development. Direct synthetic hepcidin analogs and minihepcidins (short peptides retaining the N-terminal pharmacophore) have shown preclinical efficacy in hemochromatosis and β-thalassemia models [135,136]. Antibody-based and small-molecule agonists of the BMP6 pathway are being explored for hereditary hemochromatosis [137]. In the opposite therapeutic direction, anti-hepcidin antibodies, anti-BMP6 antibodies, anti-HJV agents, ALK2 selective inhibitors, and anti-IL-6R agents have been investigated or considered for anemia of inflammation and CKD, with mixed clinical results that reflect both target validation challenges and the multifactorial nature of inflammatory anemias [72,132]. Anti-ferroportin antibodies that compete with hepcidin for binding have also entered clinical evaluation [132]. Selective ACVR1 inhibitors (developed largely in the context of diffuse intrinsic pontine glioma and fibrodysplasia ossificans progressiva) provide additional opportunities to dissect, in clinical practice, the contribution of ACVR1 to hepcidin biology in vivo and may inform second-generation MF therapies. Pacritinib, an oral JAK2/IRAK1/ACVR1 inhibitor approved for MF with severe thrombocytopenia (PERSIST-2, PACIFICA trials), shares aspects of the momelotinib mechanistic profile and produces a measurable hepcidin-suppressive effect, although it is less characterized clinically than momelotinib [102,138,139]. Together, these programs reflect a broad therapeutic interest in this axis that is unlikely to abate.

## 7. Future Perspectives

Hepcidin has emerged in the past two decades as the molecular hub through which iron homeostasis is exerted in mammalian physiology, and its dysregulation is now recognized as central to the pathogenesis of a remarkably heterogeneous spectrum of disorders, from monogenic disorders of iron loading to complex polygenic conditions such as cardio-renal anemia, the iron-restrictive anemia of inflammation, and the dual BMP–SMAD/IL-6–STAT3 hepcidin overproduction that characterizes myelofibrosis. The mechanistic dissection of the BMP6–HJV–ALK2/SMAD pathway, the identification of erythroferrone as the erythroid suppressor, the characterization of matriptase-2 as the dominant pharmacologic-grade negative regulator, and the structural elucidation of the hepcidin–ferroportin interaction together provide a remarkably coherent molecular framework for both diagnostic interpretation and therapeutic intervention.

The clinical maturation of agents acting on this axis is now substantial and accelerating. Luspatercept has substantially expanded the therapeutic armamentarium for transfusion-dependent β-thalassemia, being approved internationally for this indication, and for lower-risk MDS, where it has demonstrated superiority over ESAs in the ESA-naive setting (COMMANDS trial). Whether it should be considered a first-choice agent for all eligible patients or reserved for after a trial of standard supportive measures remains subject to regional guidelines and evolving clinical practice. Momelotinib has provided the first regulatory validation of ACVR1/ALK2 inhibition as a direct hepcidin-suppressive strategy in MF, simultaneously targeting the dual BMP–SMAD and IL-6–STAT3 drivers of MF anemia. Rusfertide is poised for first-in-class approval as a hepcidin mimetic in PV. Vamifeport and sapablursen are advancing toward potential approvals in iron overload and PV, respectively. The hepcidin axis has become, in the space of a decade, one of the most pharmacologically productive in classical hematology.

Several research priorities are emerging. First, the inter-individual variability of the hepcidin response (both under physiological conditions and across disease states) remains poorly understood, and integrative “omics” approaches combining genetic, transcriptomic, and proteomic data are likely to clarify modifier genes and environmental drivers of phenotypic expression. Second, the standardization of hepcidin and ERFE assays remains a prerequisite for clinical adoption. Third, the clinical positioning of new and approved hepcidin-targeted agents will require head-to-head comparisons with current standards of care, real-world safety surveillance (with particular attention to long-term effects on bone, the immune system, and erythroid biology), biomarker-driven patient selection, and rational combination strategies. Fourth, the therapeutic exploration of hepcidin antagonism for anemia of inflammation and CKD has so far underperformed expectations, partly because of the multifactorial nature of these conditions; refined patient stratification and combination strategies (e.g., with HIF prolyl hydroxylase inhibitors or anti-cytokine therapies) may rescue this approach, and momelotinib’s dual mechanism offers a conceptually attractive proof-of-principle for translation to non-MPN settings.

Several challenges and limitations must be acknowledged. First, patient stratification for hepcidin-targeted therapies remains poorly defined: biomarker-based selection criteria have not yet been prospectively validated, and the heterogeneity of hepcidin levels across disease states limits their predictive utility in routine clinical practice. Second, therapeutic strategies aimed at antagonizing hepcidin in anemia of inflammation and CKD have so far underperformed expectations in clinical trials, suggesting that the multifactorial nature of these conditions cannot be adequately addressed by targeting a single pathway. Third, the long-term consequences of chronic pharmacological modulation of the hepcidin–ferroportin axis remain incompletely characterized, including potential effects on nutritional immunity and susceptibility to infection, on bone remodeling via interference with the BMP pathway, and on erythroid biology beyond the intended therapeutic window. Fourth, the absence of internationally harmonized hepcidin assays continues to limit the translation of hepcidin as a clinical biomarker, despite progress in inter-laboratory harmonization efforts. Addressing these challenges will be essential to realize the full translational potential of this axis.

## 8. Conclusions

Hepcidin biology offers a paradigm of how a single peptide hormone, integrated into a hierarchical molecular network, can connect basic biochemistry to clinical medicine across multiple specialties. The maturation of this field into an actionable therapeutic axis represents a model of translational success and a reminder of how much remains to be learned at the interfaces of iron metabolism, erythropoiesis, immunity, and disease.

## Data Availability

No new data were created.
